# Overexpression of Rice *BSR2* Confers Disease Resistance and Induces Enlarged Flowers in *Torenia fournieri* Lind

**DOI:** 10.3390/ijms23094735

**Published:** 2022-04-25

**Authors:** Satoru Maeda, Katsutomo Sasaki, Hisatoshi Kaku, Yasukazu Kanda, Norihiro Ohtsubo, Masaki Mori

**Affiliations:** 1 Institute of Agrobiological Sciences, National Agriculture and Food Research Organization (NIAS), Tsukuba 305-8602, Japan; satorum@affrc.go.jp (S.M.); kakuhisatoshi@yahoo.co.jp (H.K.); kanday@affrc.go.jp (Y.K.); 2 Institute of Vegetable and Floriculture Science, National Agriculture and Food Research Organization (NIVFS), Tsukuba 305-0852, Japan; kattu@affrc.go.jp (K.S.); nohtsubo@kpu.ac.jp (N.O.); 3 JICA Tsukuba Center, Tsukuba 305-0074, Japan

**Keywords:** torenia, *BSR2*, disease resistance, genetic engineering, *Botrytis cinerea*, *Rhizoctonia solani*, flower size, ornamental crops

## Abstract

Plant pathogens evade basal defense systems and attack different organs and tissues of plants. Genetic engineering of plants with genes that confer resistance against pathogens is very effective in pathogen control. Conventional breeding for disease resistance in ornamental crops is difficult and lagging relative to that in non-ornamental crops due to an inadequate number of disease-resistant genes. Therefore, genetic engineering of these plants with defense-conferring genes is a practical approach. We used rice *BSR2* encoding CYP78A15 for developing transgenic *Torenia fournieri* Lind. lines. The overexpression of *BSR2* conferred resistance against two devastating fungal pathogens, *Rhizoctonia solani* and *Botrytis cinerea*. In addition, *BSR2* overexpression resulted in enlarged flowers with enlarged floral organs. Histological observation of the petal cells suggested that the enlargement in the floral organs could be due to the elongation and expansion of the cells. Therefore, the overexpression of *BSR2* confers broad-spectrum disease resistance and induces the production of enlarged flowers simultaneously. Therefore, this could be an effective strategy for developing ornamental crops that are disease-resistant and economically more valuable.

## 1. Introduction

Phytopathogens evade the basal defense systems of host plants to obtain specific nutrients; most of them infect only specific hosts. However, they infect different organs and tissues, depending on the host plant species. In addition, some of them infect a wide range of host plants and organs, and they cause devastating diseases in many crops. The fungus *Botrytis cinerea* causes gray mold in more than 200 plant species, including vegetables, fruit crops, and ornamental crops; it can infect many tissues, including leaves, stems, flowers, and fruits [1]. *Rhizoctonia solani*, a soil-borne fungus, infects leaves and causes sheath blight in rice [2]. In addition, it causes root rot in sugar beet and damping off in tomatoes and many ornamental crops [3] by infecting the roots. Thus, *R. solani* infects different tissues and causes devastating diseases, depending on the host plant species. A control strategy against these pathogens would be the introduction of disease-resistant genes into the host plants using the conventional breeding approach. However, there are few genes that have been found to be effective against these pathogens. Ornamental crops are considered high-quality products; therefore, they should have higher disease resistance compared to that of other crops. Ornamental crops cover many species and varieties, which makes conventional breeding more difficult in these plants. Therefore, introducing defense-conferring genes in these crops by genetic engineering could be an efficient approach.

The overexpression (OX) of rice (*Oryza sativa* L.) *BROAD-SPECTRUM RESISTANCE2* (*BSR2*) gene, encoding a cytochrome P450 (CYP78A15), confers resistance against the bacterial pathogen *Pseudomonas syringae* pv. *tomato* DC3000 (*Pst*DC3000), and the fungal pathogens *Colletotrichum* *higginsianum* and *R. solani* in *Arabidopsis thaliana* [4]. In addition, *BSR2*-OX *Arabidopsis* exhibits enlarged reproductive organs, slower growth, and extremely low seed fertility compared to that in the wild-type (WT) plants [4]. Some members of the CYP78As, such as CYP78A5, A6, and A9 influence the morphogenesis of reproductive organs [5,6,7], which could be attributed to a novel mobile growth signal distinct from that of the classical phytohormone involved in this process [5,6]. Similar to that in *Arabidopsis*, *BSR2*-OX confers resistance to two *R. solani* isolates in rice; these rice exhibit larger seeds and flowers, slower growth, and extremely low seed fertility compared to that in WT plants [4]). *BSR2*-OX tomato displays broad-spectrum disease resistance against fungi, such as *B. cinerea* and *R. solani*, and against bacteria, such as *P. syringae* pv. tomato and *Ralstonia* *pseudosolanacearum*. However, the overexpression of *BSR2* does not induce remarkable morphological changes in tomato [8]. We validated whether *BSR2*-OX can confer disease resistance and induce larger flowers in the ornamental crop, *Torenia fournieri* Lind., which is not only a model but also as a commercially distributed ornamental crop. *T. fournieri* was chosen because it has a short generation time, small size, and can be propagated using stem cuttings; therefore, it is easy to obtain plant samples in a short time. In addition, its genome size is small (2*n* = 18, 171 Mbp) [9], and tissue culture and transformation methods for this plant are established. 

## 2. Results

### 2.1. Overexpression of BSR2 Conferred Resistance against Two Fungal Pathogens in T. fournieri 

**Figure 1:**
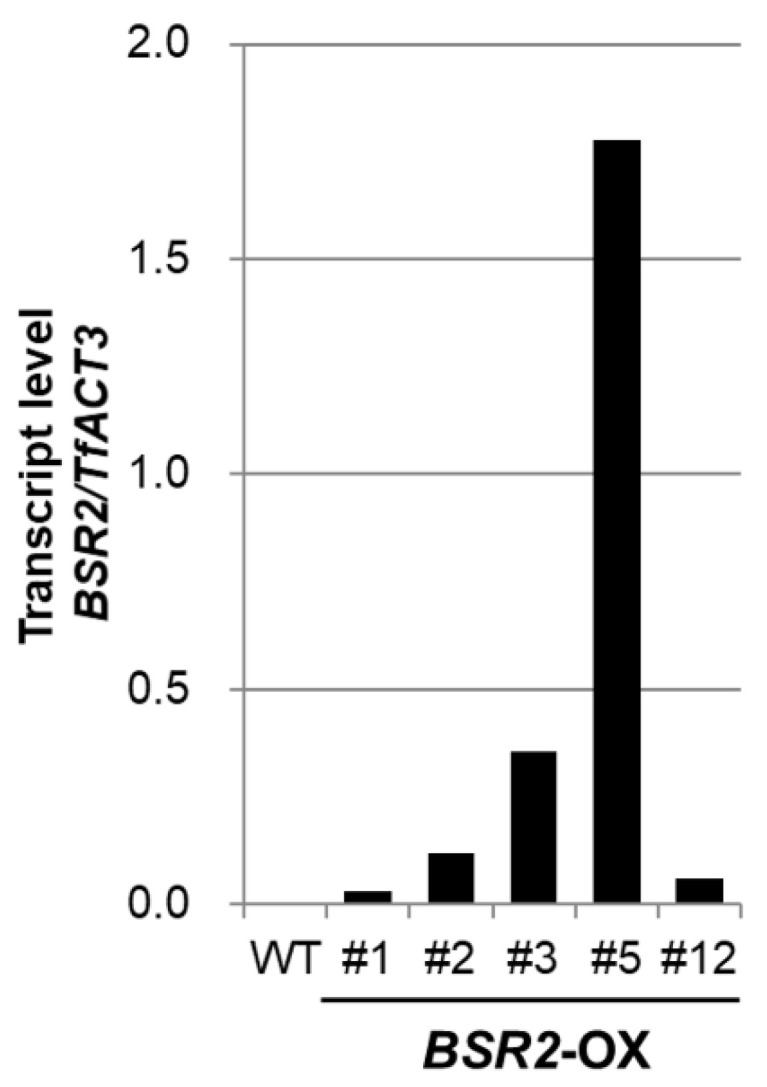
Transcript levels of *BSR2* in transgenic torenia lines.

**Figure 2:**
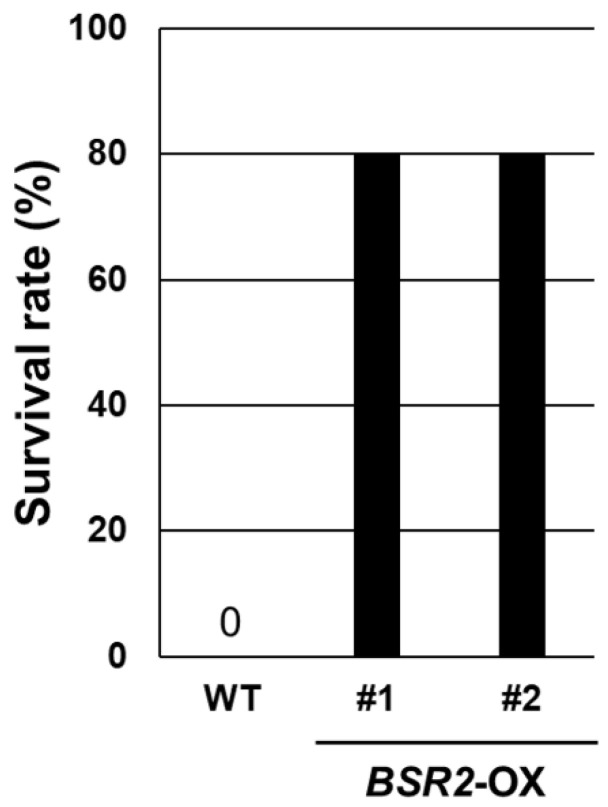
Survival ratio of transgenic torenia against *R. solani* (MAFF235116: AG-4IIIA) in the soil inoculation assay. Two-week-old *BSR2*-OX lines were transplanted into the infected soil inoculated with *R. solani*, and the survival ratios were calculated 5 days after inoculation (*n* = 5).

**Figure 3:**
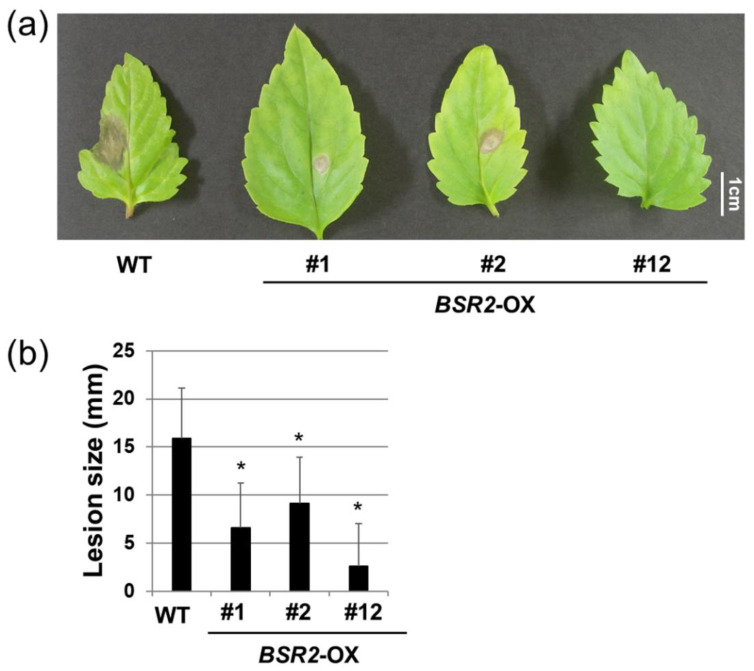
(**a**) Phenotypic changes and (**b**) lesion size of *B. cinerea* (MAFF237249) drop inoculation on leaves. Leaves of three *BSR2*-OX lines were drop inoculated with 6 × 10^3^ spore/mL of *B. cinerea* (MAFF237249). Error bars represent standard deviations (*n* = 8). * values significantly different from WT (* *p* < 0.05; Dunnett’s tests).

The *BSR2* expression vector contains rice *BSR2* cloned downstream of the constitutive 35S promoter; it was used for tomato transformation [8]. Using this vector, several transgenic *T. fournieri* lines were generated. The overexpression of *BSR2* was confirmed using quantitative RT-PCR (qRT-PCR) of independent T0 plant lines (Figure 1). Plants propagated through cuttings were used for the subsequent experiments. For the convenience of propagation, several different OX lines were used in different experiments. First, we examined whether *BSR2*-OX *T. fournieri* was resistant to *R. solani* isolate (MAFF235116; AG-4 ⅢA) infection in the roots. All WT plants died; however, 80% of the plants from the two *BSR2*-OX lines survived until five days after inoculation (Figure 2). Therefore, *BSR2* conferred resistance to *R. solani* in *T. fournieri*. Next, we evaluated whether *BSR2*-OX *T. fournieri* exhibited resistance to *B. cinerea* (MAFF237249) inoculation using the drop method [8] in leaves. Leaves of three *BSR2*-OX *T. fournieri* lines showed restricted lesion formation around the drop inoculation points; however, leaves of the WT plants developed extended lesions three days after inoculation (Figure 3a). The lesion sizes in the three *BSR2*-OX lines were significantly lesser than that in the WT plants (Figure 3b).

### 2.2. Overexpression of BSR2 Resulted in Enlarged Flowers with Enlarged Floral Organs

Flower or petal morphology is an important breeding target, because it has the most influence on the commercial value. Enlarged flowers are observed when *BSR2* is overexpressed in *Arabidopsis* and rice [4]. We investigated the size of each floral organ in the *BSR2*-OX *Arabidopsis* lines before investigating the same in the *BSR2*-OX *T. fournieri* lines. The lengths of the floral organs, the petal, sepal, stamen, and pistil, in the two *BSR2*-OX *Arabidopsis* lines at four days after flowering were 39.6–47.2%, 72.2–72.8%, 8.4–71.5%, and 100–131% longer than that in the WT, respectively (Figure S1). 

**Figure 4:**
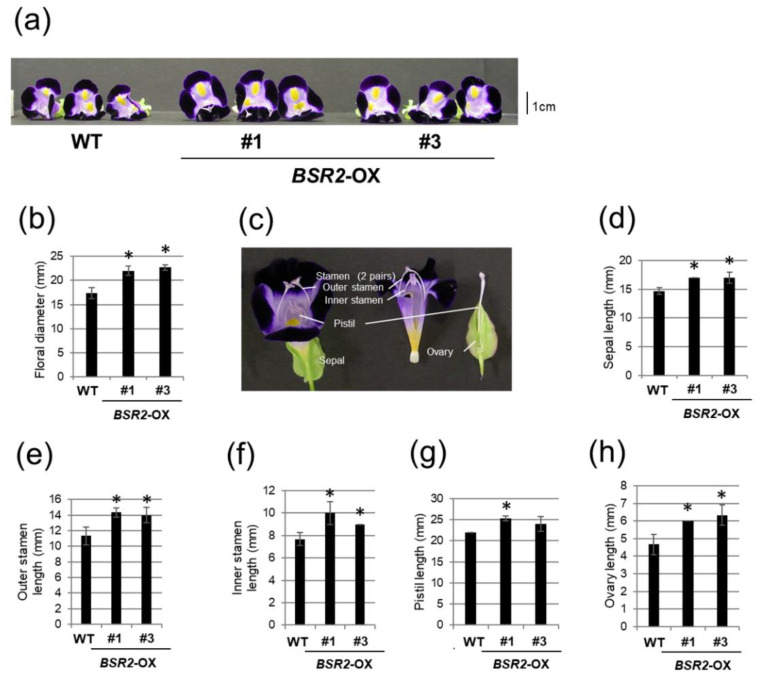
Comparison of floral morphology between WT and *BSR2*-OX torenia. Comparison of (**a**) flowers and (**b**) floral diameter between WT and *BSR2*-OX lines 6 days after flowering. (**c**) Schematic representation of torenia flower. Comparison of longitudinal lengths of (**d**) sepals, (**e**) outer stamens, (**f**) inner stamens, (**g**) pistils, and (**h**) ovaries 6 days after flowering. Error bars represent standard deviation (*n* = 3). * values significantly different from WT (* *p* < 0.05; Dunnett’s tests).

Next, we investigated the morphological traits in *BSR2*-OX *T. fournieri* lines (6 days after flowering). All *BSR2*-OX *T. fournieri* lines had larger flowers than that in the WT. The representative two lines were further analyzed. The floral diameters of the two *BSR2*-OX *T. fournieri* lines were 26.9–30.8% larger than that in the WT (Figure 4a,b). We measured the sizes of floral organs (Figure 4c). The sepal, outer stamen, inner stamen, pistil, and ovary longitudinal lengths of the two *BSR2*-OX *T. fournieri* lines were 15.9, 23.5–26.5, 17.4–30.4, 9.1–15.2, and 28.6–35.7% longer than that in the WT lines, respectively (Figure 4d–h). Therefore, the overexpression of *BSR2* enlarged floral organs in *T. fournieri*. In addition, the *BSR2*-OX *T. fournieri* plants were taller at the reproductive stage (Figure S2), similar to that in *BSR2*-OX *Arabidopsis* [4]. Enlarged flowers are often a result of polyploidization through tissue culture and transformation. Flow cytometry using 4′,6-diamidino-2-phenylindole (DAPI) was used to examine the nuclei samples of WT and the representative *BSR2*-OX *T. fournieri* lines; the relative fluorescence intensity of the nuclei was measured. The mean values at the main (2C) peak were almost similar between the WT and *BSR2*-OX lines (Figure S3a,b). In addition, a mixture of the WT and each OX line nuclei samples exhibited only one main peak (Figure S3c). Therefore, polyploidization does not occur in the tested *BSR2*-OX lines, indicating that the detected phenotypes are the result of *BSR2* overexpression.

**Figure 5:**
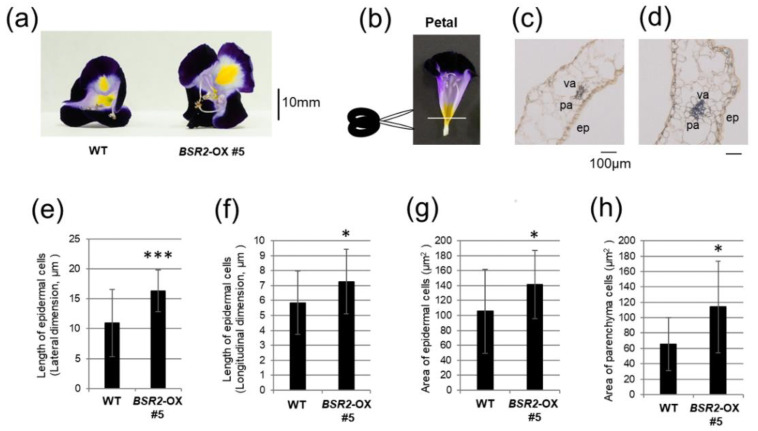
Comparison between the cellular morphology of the petal in WT and *BSR2*-OX torenia. (**a**) Comparison of flowers 7 days after flowering. (**b**–**d**) Cross-section of the basal part of (**c**) WT and (**d**) *BSR2*-OX#5 petal. ep, epidermal cell; pa, parenchyma cell; va, vascular cell. (**e**) Lateral and (**f**) longitudinal dimensions of WT and *BSR2*-OX epidermal cells in the basal part of the petal. Area of (**g**) epidermal cells and (**h**) parenchyma cells adjacent to the vascular cells in the basal part of the petal in WT and *BSR2*-OX. Error bars represent standard deviation. *n* = (**e**) 19–25, (**f**) 21–25, (**g**) 19–25, and (**h**) 10–13. * values significantly different from the WT (* *p* < 0.05 and *** *p* < 0.001, *t*-test).

Petals with a strong phenotype (Figure 5a) were examined to determine whether the cell size was affected. Sections of the basal parts of the petals, 7 days after flowering, were histologically observed (Figure 5b–d). The length and area of the epidermal cells (ep) and the area of parenchyma cells (pa) adjacent to the vascular cells (va) were measured. The lateral direction length, longitudinal direction length, and area of the *BSR2*-OX ep were 49.4, 24.3, and 34.0% longer or larger than that of the WT ep, respectively (Figure 5e–g). The area of *BSR2*-OX pa was 73.2% larger than that in the WT pa (Figure 5h). Therefore, the enlargement of the floral organs was attributed to the hypertrophy of the cells.

## 3. Discussion

In this study, we used two necrotrophic pathogens: *B. cinerea*, which causes gray mold in many aboveground organs including leaves, stems, flowers, and fruits; and *R. solani*, which causes various diseases including damping off in roots. *BSR2*-OX *T. fournieri* showed resistance to *B. cinerea* inoculated in leaves (aboveground organs) and to *R. solani* introduced in roots (underground organs), similar to that in *BSR2*-OX *Arabidopsis* and tomato [8]. Therefore, the constitutive overexpression of *BSR2* could confer disease resistance in both aboveground and underground organs. The CYP78A subfamily, including BSR2, is distributed widely in land plants including moss, *Arabidopsis*, tomato, soybean, and wheat [10,11,12,13,14]. Therefore, the BSR2 orthologs could have a function similar to that of BSR2 in plant immunity. *BSR2* encodes cytochrome P450; therefore, it would oxidize a substrate to form a “product” related to disease resistance. *Arabidopsis*, tomato, and *T. fournieri* plants overexpressing *BSR2* are resistant to two necrotrophic fungi; therefore, the “product” could be common among these plants, activating immunity and countering the potent toxins and enzymes derived from both fungi.

In *Arabidopsis*, CYP78A5(KLUH), A6, and A9 regulate reproductive organ size [6,7,15]. *CYP78A5* (*KLUH*)-OX *Arabidopsis* has enlarged flower organs because of increased cell proliferation [6]. *CYP78A9*-OX and *CYP78A6*-OX *Arabidopsis* have larger floral organs, fruits, and seeds [5,7,15]. In *CYP78A6*-OX *Arabidopsis*, there is an increase in the cell size and cell number in the reproductive organs [7]. BSR2 is homologous to *Arabidopsis* CYP78A6, A8, and A9 [4]. The size of each floral organ increased in *BSR2*-OX *Arabidopsis* and *T. fournieri*; therefore, to evaluate the change in cell size, we performed histological studies. The enlargement of the floral organs was due to the elongation and expansion of the cells; however, we could not deny the possibility of increased cell number.

The overexpression of *BSR2*, from the monocot rice, confers disease resistance against two fungal pathogens and induces enlargement (around 30% in diameter) of the flowers in the dicot *T. fournieri*. These findings encourage the use of *BSR2* for conferring broad-spectrum disease resistance and for inducing larger flowers in other ornamental crops. *Arabidopsis* CYP78A9 influences the flavanol biosynthesis pathway [15]; maize CYP78A1 exhibits lauric acid 12-monooxygenase activity [16]; and tomato CYP78A5 orthologue (SlKLUH) influences lipid metabolism [17]. However, CYP78A family members, excluding BSR2, have not been reported to be involved in disease resistance. This is likely due to a lack of research about the same thus far. The future studies should focus on identifying the secondary metabolites biosynthesized by the BSR2 protein, CYP78A15. This would enable elucidating the mechanism underlying the defense against broad-spectrum pathogens and the flower morphogenesis, and it could also lead to more efficient breeding in ornamental crops.

From an agricultural point of view, gray mold caused by *B. cinerea* results in severe economic losses in many important ornamental crops [18]. Gray mold is also a major post-harvest disease as well as a growth stage disease of roses [19]. In this study, leaves in cut branches of *BSR2*-OX *T. fournieri* plants showed *B. cinerea* resistance, suggesting that *BSR2*-OX would be effective in the post-harvest stage; in other words, protection by *BSR2*-OX might be effective in the cut flowers. Generally, commercially important ornamental crops require protection from fungal diseases in both growth and post-harvest stages. Hence, the overexpression of *BSR2* could be an attractive tool in the ornamental crops.

*T. fournieri* ‘Crown Violet’ used as a WT material in this work is derived from F1 hybrid seeds of ‘Crown Mix’; many phenotypes change in the subsequent generations due to segregation; therefore, seed proliferation of *BSR2*-OX *T. fournieri* plants was not expected from a practical point of view. Instead, vegetative propagation via cuttings would be more practical. Generally, the overexpression of *BSR2* would lead to low fertility, considering the low fertility of *BSR2*-OX *Arabidopsis* and *BSR2*-OX rice [4], although we do not have data relating to fertility in this study. However, when low fertility is not preferable, the use of a leaf-specific and/or floral organ (e.g., petal)-specific promoter [20] would be effective in maintaining fertility. The expression of *BSR2* under such mixed promoters would help confer disease resistance and induce enlarged flowers simultaneously without decreasing fertility. 

## 4. Materials and Methods

### 4.1. Plant and Microbial Materials and Culture

*T. fournieri* Lind. ‘Crown Violet (CrV)’ was used as a WT material. Violet-flowered CrV cultivar selected from F1 hybrid seeds of ‘Crown Mix’ (Sakata Seed Co., Yokohama, Japan) was kindly provided by Dr. Ryutaro Aida (Institute of Vegetable and Floriculture Science, NARO, Tsukuba, Japan). One vigorously growing F1 hybrid plant was propagated vegetatively by herbaceous cutting by Dr. Ryutaro Aida and used as the experimental line.

The WT and transgenic *T. fournieri* plants were cultured and maintained on MS medium with sucrose and/or trehalose as a carbon source, as described previously [21]. Sterile moistened black peat moss (Sakata Super Mix A) was used for *T. fournieri* soil culture. *BSR2*-OX *Arabidopsis* lines and Col-0 (WT) were grown, as described previously [4]. 

The fungal pathogens *R. solani* (MAFF235116: AG-4ⅢA) and *B. cinerea* (MAFF237249) were used to test disease resistance in *T. fournieri*. The pathogens were cultured, as described previously [8].

### 4.2. T. fournieri Transformation

In order to generate transgenic *T. fournieri* plants overexpressing *BSR2* (Os08g0547300), the recombinant binary plasmid pBIG2113SF-BSR2 [4] was used. *T. fournieri* transformation was performed, as described previously [22,23]; 20 μg L^−1^ of hygromycin-B was used for the selection. T0 plants propagated through cuttings were used for the experiments.

### 4.3. RNA Extraction and Quantitative Real-Time RT-PCR (qRT-PCR) Analysis 

Total RNA was extracted and purified from *T. fournieri* leaves using Sepasol-RNA Super G reagent (Nacalai Tesque, Kyoto, Japan) and qRT-PCR was performed, as described previously [4]. The primers used for qRT-PCR were: *BSR2:* 5′-GGACTAAGACGAGGAGAGGGAAG-3′ and 5′-AACGTAGGGGCATTTCTACTCAA-3′; *TfACT3:* 5′-AAGATATGCATTGGAGTTGTGAGTG-3′ and 5′-TCCAAGCTAAGGTAGCAAAACGA-3′. 

### 4.4. Fungal Pathogen Resistance Assay 

An inoculum suspension of *R. solani* was prepared as described previously [8] and mixed with sterile moistened black peat moss (Sakata Super Mix A). Plants were cut about two weeks before inoculation; rooting was promoted in water. They were transferred to the infected soil and grown under long-day conditions (16 h light and 8 h dark) at 25 °C for 5 days; survival ratios were calculated. An inoculum suspension of *B. cinerea* (6 × 10^3^ conidia/mL) was prepared, as previously described [8]. Vegetative branches with one node and leaves were cut from *T. fournieri* plants, and then, the cuttings were inserted into the floral form with water. *B. cinerea* inoculum (5 µL) was dropped on the leaves of the cut branches. The inoculated branches were incubated under humid, long-day conditions, at 25 °C, and the lesion sizes were measured three days after inoculation.

### 4.5. Flow Cytometry

Flow cytometry using a Ploidy Analyser/Flow cytometer (Partec GmbH, Munster, Germany) was performed, as described previously [24,25]. The staining solution contained 10 mM Tris, 2 mM MgCl_2_, 0.1% (*v*/*v*) Triton X-100, and 2 mg L^−1^ DAPI at pH 7.5. 

## Data Availability

Data are contained within the article.

## Supplementary Materials

The following are available online at https://www.mdpi.com/article/10.3390/ijms23094735/s1.
