# Supplementary material for: Overexpression of Rice BSR2 Confers Disease Resistance and Induces Enlarged Flowers in Torenia fournieri Lind
Source: Int J Mol Sci. 2022 Apr 25;23(9):4735. doi: 10.3390/ijms23094735 (PMC9102792; doi:10.3390/ijms23094735)
Supplement: Supplementary file 1 [file ijms-23-04735-s001.zip › ijms-1675763-supplementary.pdf]

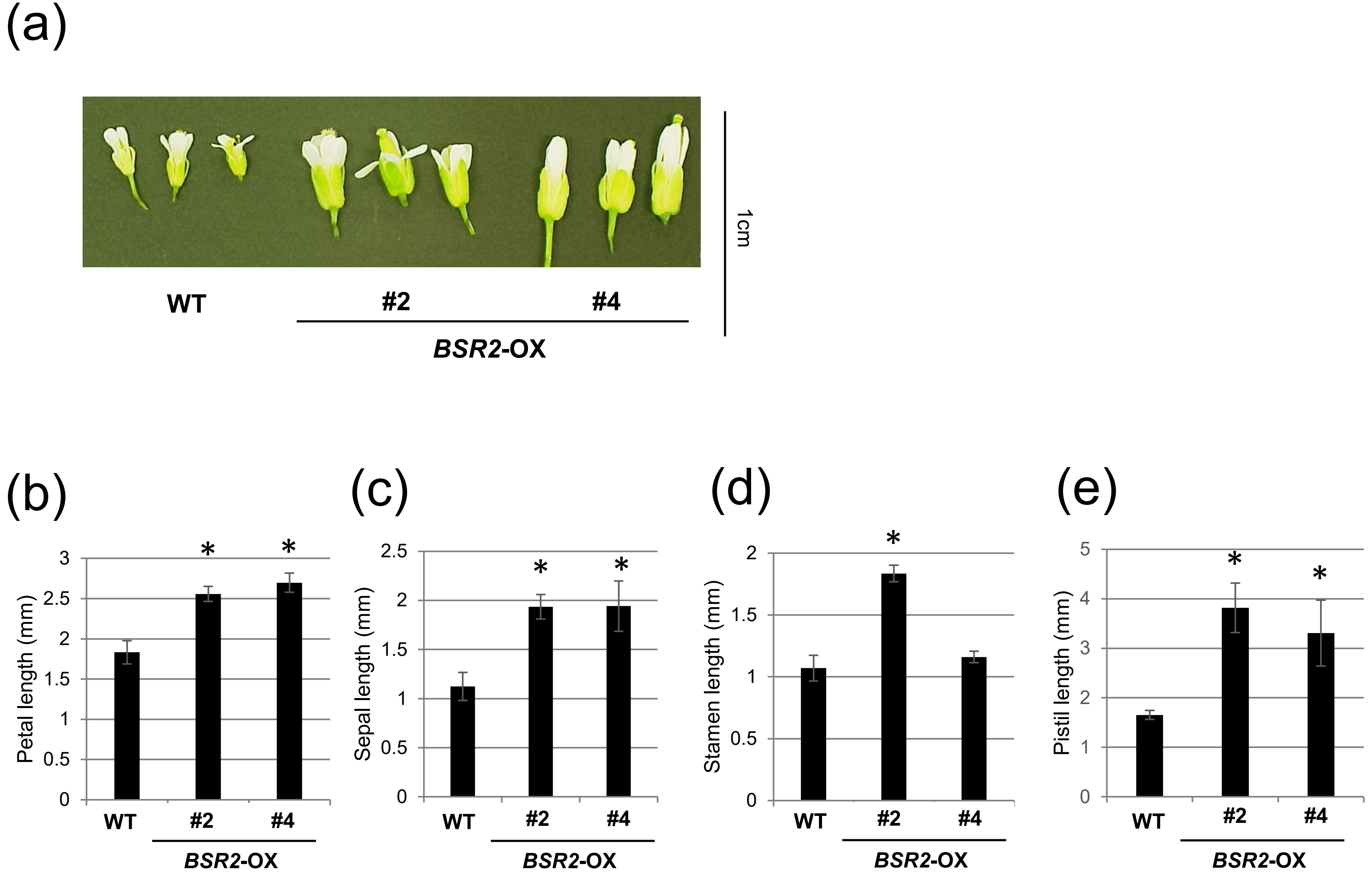

Figure S1. Comparison of flowers and floral organs between WT and *BSR2*-OX *Arabidopsis* lines. (a) Comparison of flowers between WT and *BSR2*-OX lines, 4 days after flowering. Comparison of longitudinal lengths of (b) petals, (c) sepals, (d) stamens, and (e) pistils, 4 days after flowering. Error bars represent standard deviations (n = 4–6). *values significantly different from that in WT (*P<0.05; Dunnett's tests).

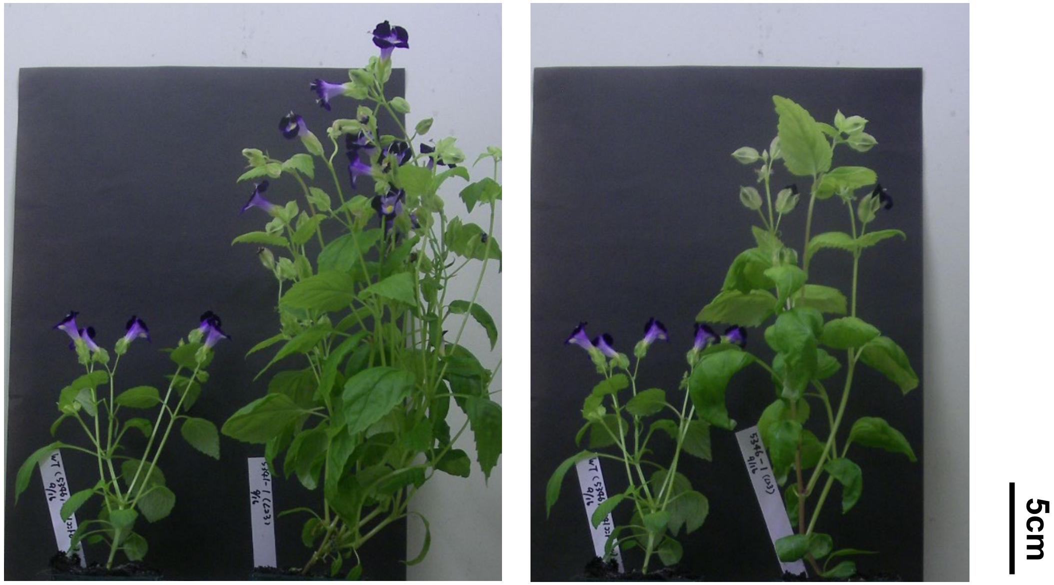

Figure S2. Comparison of adult whole plants 70 days after propagation by cutting.

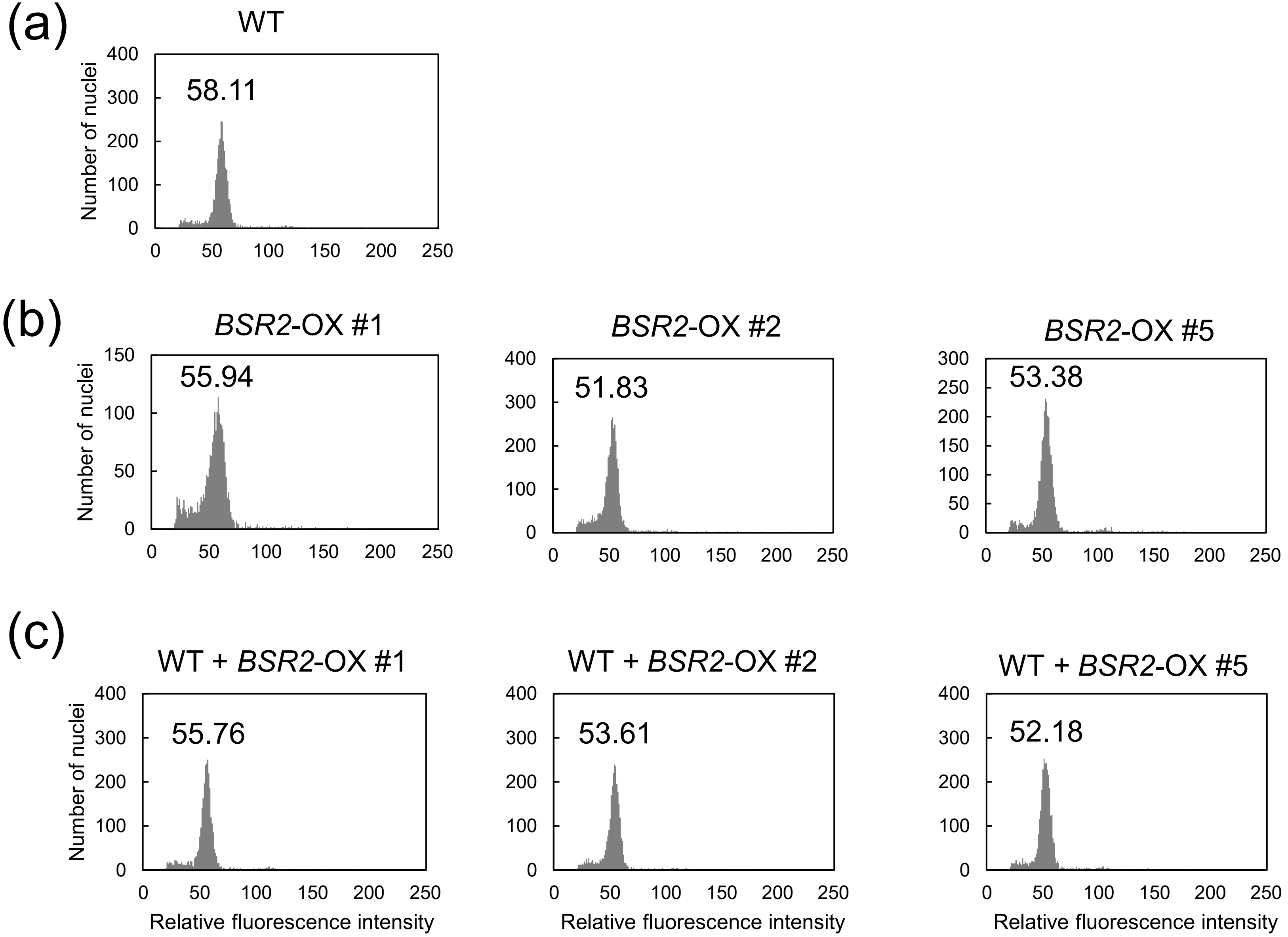

Figure S3. Typical flow cytometry histograms for nuclei isolated from leaves of torenia. (a)WT. (b) Representative *BSR2*-OX lines. (c) Mixture of an equal amount of WT and representative *BSR2*-OX lines. Number represents the mean value at the main (2C) peak.
